# Why does gender matter for immunization?^☆^

**DOI:** 10.1016/j.vaccine.2022.11.071

**Published:** 2024-04-08

**Authors:** Goodman Tracey, Bullock Olivia, Munro Jean, Holloway Megan, Singh Sagri

**Affiliations:** a Department of Immunization, Vaccines and Biologicals (IVB), World Health Organization, Geneva, Switzerland; b Gavi the Vaccine Alliance, Geneva, Switzerland; c UNICEF, NY, USA; d Independent Consultant, Geneva, Switzerland

**Keywords:** Vaccines, Gender, Equity, IA2030, Immunization, Vaccination

## Abstract

Attention to gender-related issues in immunization programmes goes beyond focusing on coverage discrepancies between girls and boys. There are multiple ways in which gender roles, norms and relations influence resource allocation, decision making, access, and health outcomes, including for immunization programmes. Gender impacts immunization both on the demand side through people’s health seeking behaviours, and the supply side through provision of health services. To increase immunization coverage, and in particular to reach zero-dose children, it is necessary to understand and address the many ways in which gender interacts with additional socio-economic, geographic and cultural factors -- such as age, race/ethnicity, religion, marital status, education, wealth, sexual orientation and gender identity, HIV status, disability and migration status -- to influence access to and delivery of vaccines. The Immunization Agenda 2030 (IA2030) commits to addressing gender-related barriers to immunization and advancing gender equality and gender is an important cross-cutting consideration for all seven IA2030 strategic priorities. Gender-responsive strategies are particularly highlighted as an IA2030 key area of focus for Strategic Priority 3: Coverage & Equity. Gender-related barriers and gender inequality can prevent people, both male and female and those of diverse gender identities, from getting vaccinated. These operate at multiple levels from the individual and family/household to community and within institutions/systems and national policies/frameworks and are underpinned by power relations leading to different opportunities, limitations, challenges, needs and vulnerabilities, especially for women and girls. By applying knowledge about gender and taking action to design gender-responsive interventions, it is possible to implement more effective immunization programmes and increase coverage for all.

## 1 Introduction

Gender and equity are cornerstones of Universal Health Coverage (UHC) and Primary Health Care (PHC). Addressing gender-related barriers and the specific needs of women and men in health services, advancing gender equality, and increasing women’s meaningful participation in the design and delivery of health services are key to advance the Sustainable Development Goals (SDGs) and to achieve UHC [1].

There are multiple ways in which gender roles, norms and relations influence who gets sick, who has access and control over resources, who’s voice and decisions are heard, and who’s health needs are prioritized and met [2] including for immunization. In immunization programmes, gender-related barriers go beyond concerns of coverage discrepancies between girls and boys [3]. Gender interacts with multiple socio-economic, geographic and cultural factors -- such as age, race/ethnicity, religion, marital status, education, wealth, sexual orientation and gender identity, HIV status, disability and migration status -- to influence access to and delivery of vaccines. As such, to increase immunization coverage and in particular to sustainably reach zero-dose children and missed communities, it is necessary to understand and address the many ways in which gender impacts immunization programme performance both through the demand for, and supply/provision of, vaccination services as part of primary health care.. Applying a gender lens to increase immunization coverage further contributes to achieving gender equality and empowering all women and girls (SDG 5) [4].

## 2 Immunization Agenda 2030 and gender

**Fig. 1:**
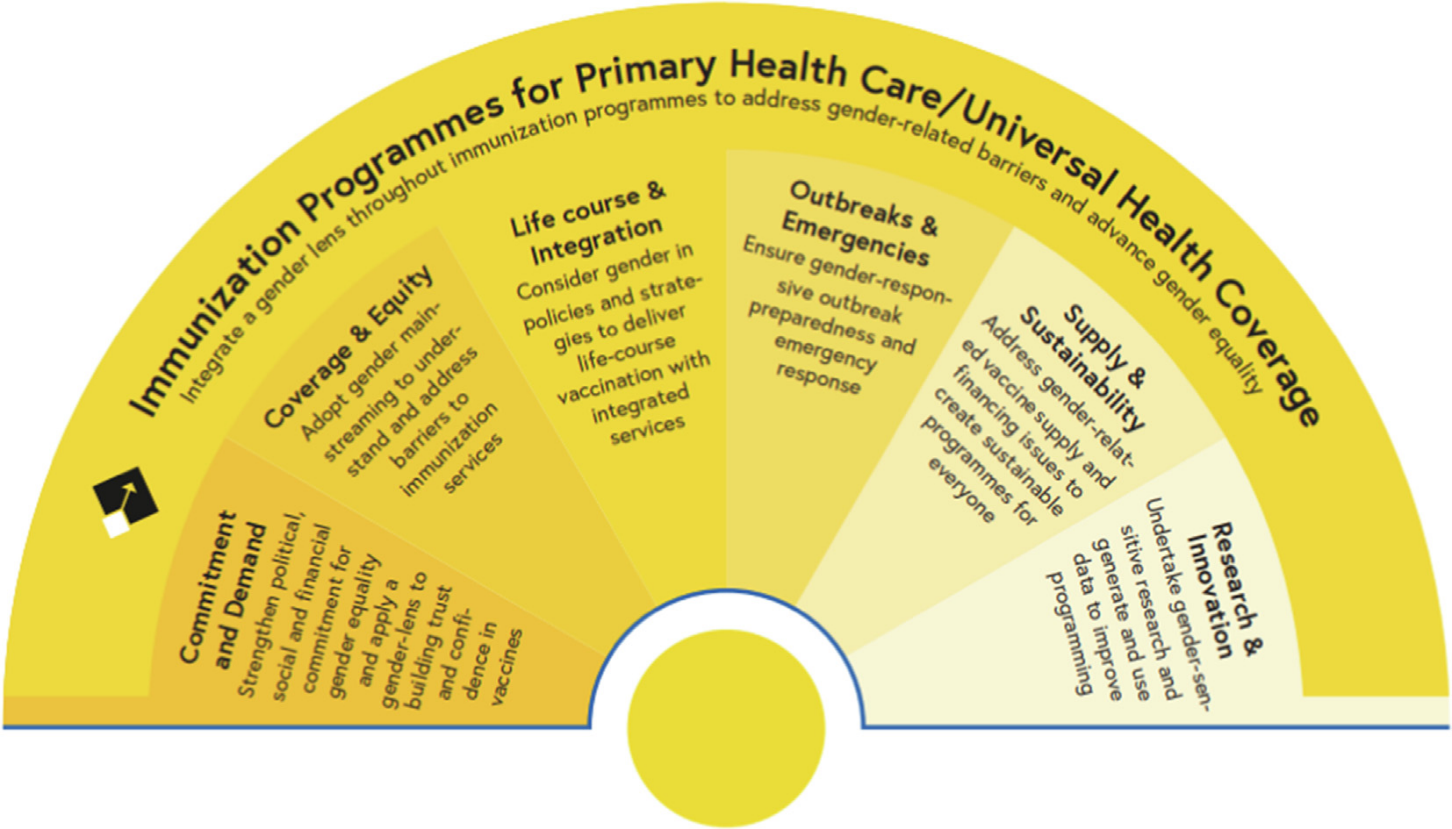
Gender and the Immunization Agenda 2030 Strategic Priorities.

The Immunization Agenda 2030 commits to addressing gender-related barriers to immunization and advancing gender equality in order to realize its vision for the decade: A world where every-one, everywhere, at every age, fully benefits from vaccines for good health and well-being. Under the Strategic Priority Area #3: Coverage and Equity[5], IA2030 focuses on ensuring equitable vaccination coverage of girls, boys, women and men, and includes those of gender diverse identities. However, as Fig. 1 illustrates, gender is an important cross-cutting consideration for all seven IA2030 strategic priorities.

IA2030 aims to address direct and indirect barriers to immunization services shaped by gender and gender inequality, including demand related barriers e.g., access to health information, resources, decision-making, and people’s experiences of health services, as well as supply-related barriers, such as government policies, hiring, remuneration and promotion practices of the health workforce, and quality of services and health worker attitudes. Identifying and addressing these barriers can be done through employing gender-responsive strategies, which consider the different needs, experiences, vulnerabilities of women, men, girls and boys – including gender-transformative actions that aim to dismantle harmful gender norms and roles, empower women and advance gender equality.

The seven strategic priorities of the IA2030 are anchored in the four core principles below. Viewed through a gender lens, these principles express gender equality as a fundamental value, and provide guidance for the translation of the high-level IA2030 strategy into practical actions.

**Table:** 

**Panel: IA2030 Core Principles and Gender**
**People-focused: Responding to the different needs of women, men, girls and boys**
The design, management and delivery of immunization services should be shaped by and responsive to the needs, experiences, preferences and vulnerabilities of women, men, girls and boys of diverse backgrounds. Programme design should be led by and inclusive of communities that programmes are targeting (e.g. marginalized or missing communities where zero dose children are often found). Community based approaches should be favoured over one-size fits all national or regional solutions. When programmes target women, women should lead programme design.
Do no harm should be applied as a central principle in programming to avoid creating adverse impacts or reinforcing harmful gender norms and roles contributing to marginalization, sexual harassment, exploitation and other forms of gender-based violence.
**Country-owned: Driving progress through country gender equality commitments**
Country ownership and accountability should be promoted at all levels, ensuring that countries recognise the potential and importance of addressing gender-related barriers to accessing and utilizing healthcare, and that they are equipped with the resources to identify and address gender-related and other intersecting social barriers in health services.
Developing partnerships with relevant gender equality processes and commitments, including the SDGs (particularly SDG5) at the country level is key. This includes relevant community-based organizations, civil society, Ministries of Gender/Women, Finance and other line ministries, national committees responsible for driving results for SDGs and other national and global gender equality commitments. Immunization programmes should be targeted and tailored based on country context, rooted in an understanding of context-specific gender norms, roles and relations and their links with health outcomes. Women’s equal participation in national immunization bodies and advisory groups must be promoted.
**Partnership-based: Aligning efforts to maximize impact for gender equality.**
Immunization partners, including ministries of health should align and coordinate their actions to increase efficiency, build on complementarity and involve sectors beyond health for mutual benefit in identifying and addressing gender-related barriers to immunization, and increasing women’s meaningful participation at all levels of the health system.
Local communities and civil society, especially women’s and adolescent girls/youth led groups and organizations, should be engaged and involved in planning, implementation and oversight of immunization interventions to strengthen accountability and sustain impact. Other critical partners include national ministries of Gender/Women, Gender units in health ministries and global partners such as UN Women, UNFPA, UNICEF, UNDP and other UN agencies with gender units.
**Data-guided; Promoting evidence-based decision-making informed by sex-disaggregated data and gender analysis.**
High-quality, “fit-for-purpose” gender data, disaggregated and analysed by sex and age, and additional factors such as ethnicity, disability and geographical location, should be used to track progress, identify gaps and challenges, improve programme performance, and form the basis of programming and decision-making at all levels. Particular efforts should be made to generate evidence and data via qualitative studies and behavioural science inquiry.

## 3 Mainstreaming gender in immunization programmes

Gender mainstreaming is a strategy to reach gender equality. The goal of gender equality is to ensure that every-one has equal opportunities to access immunization services and benefit from being fully vaccinated.

Gender mainstreaming involves integrating the concerns and experiences of women as well as men, into the design, implementation, monitoring and evaluation of immunization policies and programmes with a view to promoting equality and not perpetuating inequality. It requires assessing the implications for women and men of any planned action, including legislation, policies or programmes, in all areas and at all levels. It is also about understanding the ways in which the different roles and expectations within a society dictate what it means to be man and woman and subsequently, how this shapes context and the situation in which immunization programming is conducted [6]. By considering the impact of gender, more effective immunization programmes can be implemented to increase coverage.

Gender-related barriers and gender inequality can prevent people, both women and men, from getting vaccinated. These barriers operate at multiple levels from the individual and household to community and health systems and policies, and are compounded by other dimensions of inequality, all of which underpinned by unequal power dynamics leading to different opportunities, limitations, challenges, needs and vulnerabilities, especially for women and girls.

In this section, some of the most common gender-related barriers to immunization are described.

### 3.1 Barriers from poor quality services and negative health provider attitudes

Factors related to the quality, acceptability and accessibility of health services may deter women and men from attending immunization services for themselves and their children in different ways. This can include the following:

• **Poor working conditions and lack of supportive supervision:** Health workers often are performing their jobs without adequate remuneration, equipment/transport or supervision. This can create an overburdened and demoralized workforce which in turn affects the ability to provide quality services.
• **Patronizing and disrespectful treatment**: Negative experience of poor treatment by healthcare providers can influence women to stay away from health centres, resulting in a missed opportunity for vaccination. In many settings, it is unusual for men to bring children for vaccination, and they can sometimes be ridiculed, discouraging them from returning and possibly resulting in further missed vaccinations.
• **Absence of female healthcare providers:** In areas where socio-cultural and/or religious norms and practices restrict women’s mobility and social and physical contact between men and women, women may not seek care for themselves or their children unless they have access to a female health provider. In Afghanistan, for instance, a study found that women refused life-saving tetanus toxoid from male vaccinators. [11]
• **Discrimination** in healthcare settings is widespread and takes many forms, affecting both users of health services and healthcare workers (e.g. pay gaps, sexual harassment, limited promotion opportunities, etc). Although research on links with immunization outcomes is limited, research shows LGBTQI communities face significant social stigmatization, discrimination and marginalization [7], experience limited access to essential, quality and affordable health services, contributing to negative health outcomes [8]. A U.S. national survey found 28 % of trans people had postponed health care due to discrimination, and 28 % reported being harassed by health workers when they did seek care [9].
• **Disabilities** (physical and cognitive): People living with disability are more than twice as likely to report finding healthcare provider skills inadequate to meet their needs, four times more likely to report being treated badly, and nearly-three times more likely to report being denied care.[10] The intersectional impact of gender and disability may result in women and men living with a disability facing different challenges and barriers. For example, a man living with a disability may be more accepted by a community and less restricted, than a woman who may be treated as an outcast. These realities threaten to undermine universal health coverage and can impact mothers’ and fathers’ access to immunization services for their children.
• **Other service quality barriers**: Both men and women reported that the lack of separate waiting areas inhibited women’s use of health facilities.[11] Lack of adolescent-friendly services, long distances to health facilities, long waiting times, lack of appropriate opening hours, sanitation facilities, and confidentiality. These factors can have a differential impact on women and men’s access to immunization services.

### 3.2 Low education level and health literacy as a barrier

There is a strong link between maternal education, child health and positive immunization outcomes [12], [13], [14]. While lower literacy levels and lack of access to health information can hamper both women’s and men’s knowledge of, utilization and access to immunization, women are often more disadvantaged. Although fathers’ education level is also associated with a child’s immunization status, mothers’ lower educational level is more commonly related to under-vaccination [15]. Women who are more literate, regardless of their educational level, are more likely to vaccinate their children in both urban and rural settings [12].

Part of the strong link between mothers’ education level and childhood immunization coverage is explained by socio-economic status and context-specific factors, since more educated mothers tend to live in more affluent households and in areas with better access to health services [16], [17]. Children of younger women without education, especially those belonging to poor households, are more disadvantaged [18].

### 3.3 Barriers due to limited autonomy in decision-making and household dynamics

The different social roles assigned to women and men affect the degree to which women have access to, and control over decision-making related to their health, and their children’s health [19]. While traditional gender norms and roles generally render women the designated caregivers for children, men often act as decision-makers within households due to unequal power relations and gender inequality. Yet, vaccination interventions often only target women caregivers, neglecting the influence men have over decisions about immunization and other healthcare issues. Healthcare-related decision-making is negotiated (or not) within the household and extended family. Mothers may be limited in their bargaining power in both the gendered (with the male head) and generational (with elderly women and men) power dynamics of the household [20], [18].

Women’s decision-making and agency have been strongly associated with children’s immunization status. For example, a study in Nigeria showed that the higher women’s autonomy and decision-making capability, the more likely they were to immunize their children [21]. Studies in South Asia have shown that women’s greater decision-making autonomy translates into greater use of maternal and child health services and positive health outcomes [22].

Mothers who perceive that spousal permission is required for their child’s immunization are less likely to fully immunize their child. An assessment carried out in Afghanistan shows that many women cannot take independent decisions on their own health and often are culturally required to be accompanied when seeking health services. Male heads of households (i.e. husband, father or brothers) generally make those decisions for women, inhibiting timely access to health care services for women and their children [23]. This delay is further exacerbated by needing to arrange for a chaperone, once permission is granted.

### 3.4 Lack of access and control over resources and mobility as a barrier

Women, especially in low income or emergency settings, tend to have poorer access to, and control over, critical resources such as **time, money, and transportation**, that can influence health and immunization outcomes.

**Time** is a resource and an opportunity cost which can create barriers for immunization, especially for women if distances/waiting times for vaccination are very long [24]. Women are generally expected to take care of household work and caretaking responsibilities, social tasks and other family obligations, collecting water and firewood, and cooking food. This applies also to the mostly female health workforce who often struggle with multiple responsibilities and long “double” work days. Globally, women perform three times more unpaid care and domestic work than men [25]. Studies in Bangladesh, China and Gabon have pointed to time constraints limiting opportunities to health seeking and access to health services [24]. Time costs due to weak infrastructure are greatest in remote areas, while workforce participation creates additional time and income constraints for women in urban areas [18].

Although immunization services are usually free of charge, **transportation** adds to a **“hidden” cost** in many settings. In poor areas, mothers need to raise the necessary resources, or mobilize means of transport to take her child to vaccination. In a study conducted in Nigeria, the most commonly reported barrier to accessing immunization was the lack of financial resources for the costs of transportation or services [26].

Mobility may also be restricted by safety and security concerns which are heightened in conflict and emergency settings, where the prevalence of gender-based violence (GBV) generally increases [4]. In a study on women’s healthcare access in Afghanistan, almost one-third of women reported insecurity as a reason for not visiting the health facility when sick [27]. Health workers also face the same issue.

Religious or cultural norms also influence women’s mobility as they may influence interactions expected or permitted between women and men. For example, in the Nigerian Hausa tradition, unrelated men may not speak to women without permission from their husbands. In polio eradication activities, deploying women frontline workers has increased the effectiveness of immunization delivery, as in many settings only women can access households and vaccinate children inside [28]. All-male vaccinator teams, were found to be ineffective, posing a critical gender-related barrier to polio eradication efforts [29].

Women’s restricted access to and control over financial resources makes it less likely that their children are immunized. A study in Ethiopia [30] found women who had power to decide about use of financial resources were more likely to have their children partially or fully immunized, compared to women who were not involved in decision-making. Importantly, women who made financial decisions jointly with their husband were even more likely to have their children vaccinated, compared with women who made financial decisions independently, pointing to the important role men can play in improving children’s health outcomes. This signals the importance of having immunization messaging that targets the couple, as well as men and women individually.

### 3.5 Barriers created by high prevalence of gender-based violence and harmful practices

One in three women experience gender-based violence (GBV) in their lifetime [31]. Moreover, conflict and displacement may exacerbate existing violence and lead to new forms of violence against women [32]. While men and boys can also experience GBV, women and girls are disproportionately affected. Apart from being a serious human rights violation rooted in gender inequality, GBV has negative physical, psychological, sexual and reproductive health implications. Immunization services can provide critical entry points for health workers to refer GBV survivors to receive appropriate support. Health workers themselves can be subjected to GBV through sexual harassment by co-workers/supervisors and the community.

In addition, GBV also has a harmful effect on immunization outcomes. GBV may decrease the likelihood of women utilizing health services for themselves or their children due to fears of disclosing violence to others outside the household. A study focusing on the impact of intimate partner violence in childhood immunization in Bangladesh confirmed a strong and significant effect on children’s immunization levels [33]. In Rwanda a study on GBV care found that women abstained from seeking healthcare to avoid more violence and abuse and to “protect the reputation” of their husband and family [34]. GBV has been shown to contribute to overall lower use of reproductive and maternal health services and adverse child health outcomes [35]. Other related issues include:

• **Child marriage**, a form of GBV, also has a direct impact on girls’ and women’s access to and utilization of health services, including immunization. Currently, in the least developed countries 40 % of girls are married before the age 18 and 12 % of girls are married before age 15 [36]. Adolescent girls forced into child marriage are less likely to have knowledge about health, and are more likely to suffer from GBV, unwanted pregnancies and maternal morbidity and mortality, while gender norms restrict their decision-making and agency in the family and their mobility, hindering their access to vaccination services [37]. Child marriages are heavily associated with low education levels, as girls who are denied educational opportunities are more likely to marry young. Child marriages weaken girl’s and women’s negotiating power on health decisions – for example in South-West Asia, child marriages were found to lead to 11 % fewer antenatal visits; in Sub-Saharan Africa the probability of children receiving basic vaccinations was found to be twice as high and their neonatal mortality reduction nearly double if their mothers married between ages 15–17 instead of at ages 10–14 [38].
• **Son preference**, rooted in gender inequality, has also been found to decrease the likelihood of girls receiving vaccinations in some settings. Although globally there are no major disparities with girls’ and boys’ immunization coverage, the preferential treatment of boys is perpetuated in some contexts. Girls have faced greater abandonment and neglect in many areas of the world, particularly Asia [39]. Indian girls, for example, were found to be less likely to receive healthcare, have less money spent on them for medicine, and taken to health-care facilities at later stages of illness [40].

## 4 Gender-responsive approaches to increasing immunization coverage

The approaches developed and used to tackle gender barriers can be categorized along a continuum.

Programmes, policies and interventions are “gender-responsive” when gender roles, norms and inequalities have been analysed and appropriate measures have been taken to actively address them. Gender-responsive programmes can be either “gender-specific” in that they target a specific group of women or men, but do not challenge gender norms and roles, or they can be “gender-transformative” when they address the root causes of gender inequality and attempt to transform harmful gender norms and roles.

**Fig. 2:**
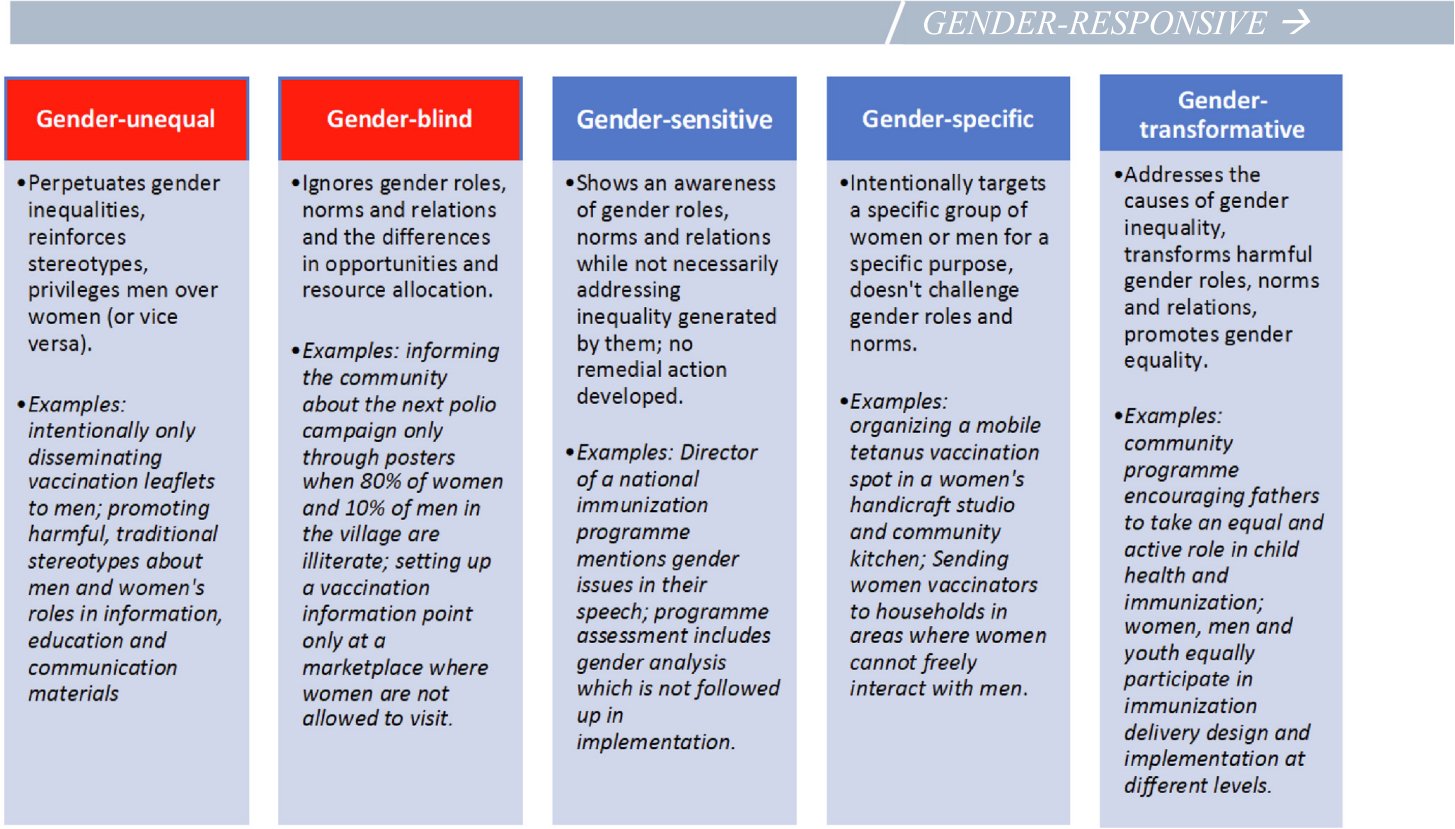
Gender-responsive assessment scale.

Immunization interventions should, at a minimum, be gender-specific, and ideally and when possible, gender-transformative. Fig. 2 adapted from WHO’s Gender-responsive Assessment Scale (GRAS) [41], provides an overview of each level, with illustrative examples related to immunization programming. Gender-unequal and gender-blind in red are harmful and contrary to the healthcare principle of *‘do no harm’*.

A new WHO/UNICEF/Gavi guide entitled ‘Why Gender Matters: Immunization Agenda IA2030′ [44] lays out in detail key gender-responsive approaches and critical actions for mainstreaming gender in immunization programmes and policies. Table 1 provides a high-level summary excerpt from this guide.

**Table 1:** Summary of gender-responsive immunization programme areas and actions.

**Immunization Gender-responsive interventions**	**Actions**
Invest in gender data and analysis	• Make sure all immunization action plans, strategies, policies, surveys and programme updates contain sex-disaggregated data and are informed by a gender analysis • Monitor gender-sensitive indicators in immunization plans and programmes
Make community engagement and social mobilization gender-responsive and transformative	• Include gender in any social mobilization situation analyses, assessments and communication plans • Ensure gender-balanced social mobilization and community engagement teams • Design immunization materials, messages and interventions to challenge harmful gender norms, roles or stereotypes. For example, portray women as equal and active participants, not only as mothers and caregivers
Engage with men to transform gender norms	• Target both men and women in all immunization outreach and messaging • Train health personnel to positively encourage men in prenatal consultations and primary health clinics to take part in children’s health and strengthen positive attitudes towards men visiting health centres with their children. • Use male influencers to model gender equality behaviours.
Empower and collaborate with civil society and change agents	• Identify and invite change agents, including women’s, men’s and youth groups and informal grassroots organizations, to participate in the planning, delivery, and monitoring and evaluation of immunization services (especially in areas with low coverage) • Partner with initiatives that aim to build women’s capacity and self-efficacy (e.g. skills building, economic empowerment) to advance gender equality, women’s autonomy and empowerment
Implement gender-responsive actions for the health workforce	• Provide safe and decent working conditions, including equal pay, supportive supervision and protection from sexual harassment and violence at work, for women in the health sector • Put in place confidential feedback/complaints mechanisms for immunization service providers and clients • Remove discriminatory human resource policies and practices to enable professional advancement of women health workers. • Increase women’s representation at all levels of decision-making and monitoring local, national and global health systems
Improve the quality, accessibility and availability of services	• Train vaccinators and healthcare workers to be respectful, responsive and empathetic to the needs and experiences of women, men, and youth, and those who may be stigmatised and marginalized. • Schedule immunization services at more appropriate/flexible times and in convenient locations for caregivers. • Provide travel vouchers and related in kind support to facilitate easier access to health services
Integrate services and collaboration with other sectors	• Integrate immunization services in other health, education, social protection or other service programmes that communities trust and fully participate in • Support and link with birth registration/civil registration and vital statistics (CRVS) systems through immunization to ensure both boys and girls births are officially recorded
Implement gender-responsive immunization services in emergency settings	• Select equal numbers of men and women for outbreak/disaster assessment teams to ensure access to all people. Where feasible, include a gender and protection specialist in the team • Use the UN Inter-Agency Standing Committee’s (IASC) Gender in Humanitarian Action Handbook [42] and the IASC Guidelines for Integrating Gender-Based Violence Interventions in Humanitarian Action [43] • Provide safe access points for vaccination, with more dispersed vaccination sites with shorter travel distances, or places where women frequent • Link immunization to sexual and reproductive health and rights (SRHR) and violence protection/mitigation programmes and services
Build commitment and ensure accountability	• Build commitments to gender equality with visible leadership and develop a unified voice on gender issues through the strategic use of gender champions at the global, regional, national and sub-national and community levels • Integrate gender specific criteria in evidence reviews of regional and national immunization technical advisory committees (RITAGs & NITAGs) • Ensure gender-balanced representation on all such advisory committees • Include gender equality in the National Immunization Strategy (NIS) and make reporting against gender equality commitments (e.g. SDG 5) an obligatory part of regular immunization progress reports and updates
Apply a gender lens to research and innovation	• Ensure that vaccine trials and roll out are designed to facilitate participation of women, men and non-binary people, including specific groups such as pregnant and lactating women • Strengthen local capacity to conduct implementation research to identify interventions that enhance coverage and equity and support tailored solutions to address gender-related inequities and barriers

## 5 Conclusion

Gender equality drives better outcomes for all people everywhere. To make progress in line with the IA2030, policymakers and practitioners cannot ignore the importance of mainstreaming gender across the immunization programme cycle – from the policy, management and design of immunization systems through to the implementation, monitoring and evaluation of the services. Adopting a gender perspective in all steps towards the goals of IA 2030 must be ensured, at all levels. Without truly gender-responsive action for immunization, our goals will remain elusive.

Successful gender mainstreaming requires changes within all organizations that implement and support immunization programmes. We must *“walk the talk”* for gender equality and ensure that our own internal policies and systems reflect this value in practice. Through our joint efforts we can realize the vision set in the *Immunization Agenda 2030* - a world where every-one, at every age fully benefits from vaccines for good health and well-being.

## Funder

World Health Organization

## CRediT authorship contribution statement

**Goodman Tracey:** conceptualization, supervision, Writing - original draft, reviewing, editing. **Bullock Olivia:** original draft. **Munro Jean:** Writing - reviewing, editing. **Holloway Megan:** Writing - reviewing, editing; **Singh Sagri:** Writing - reviewing, editing.

## Declaration of Competing Interest

The authors declare that they have no known competing financial interests or personal relationships that could have appeared to influence the work reported in this paper.

## Data Availability

No data was used for the research described in the article.
